# Reply to Farmakiotis et al

**DOI:** 10.1093/cid/ciu561

**Published:** 2014-10-15

**Authors:** Claire S. Waddington, Thomas C. Darton, Brian Angus, Andrew J. Pollard

**Affiliations:** 1 Oxford Vaccine Group, Department of Paediatrics, University of Oxford; 2 National Institute for Health Research Oxford Biomedical Research Centre; 3 Nuffield Department of Medicine, University of Oxford, United Kingdom

To the Editor—Our recent development of a human *Salmonella* Typhi challenge model provided a detailed description of the clinical profile and associated pathological changes seen during typhoid fever [1]. Alterations in hematological parameters included decreases in hemoglobin and hematocrit in all participants, and, in those diagnosed with typhoid, decreases in both platelet and total white cell counts (WCCs). The reduction in WCC manifested as falls in neutrophil and eosinophil count, with the eosinophil count declining from 6 days prior to diagnosis. This was the earliest of all the blood parameters to change and was seen in all but 1 participant.

Eosinopenia in our study was part of a general suppression of hematopoiesis, possibly consequent to infiltration of the bone marrow by *Salmonella* Typhi. As Farmakiotis et al note, the finding of eosinopenia has previously been reported in typhoid, although not universally. For example, in 2 series of 28 and 54 children with typhoid, eosinopenia occurred in 71.4% and 86.6%, respectively [2, 3]. Similarly, 10 of 17 returning travelers with typhoid fever were eosinopenic [2]. These data call into question the diagnostic value of the eosinophil count in isolation, and highlight the need to assess the clinical picture in its entirety. Clinical diagnosis of typhoid fever is challenging, particularly in endemic settings where the presentation can be indistinguishable from other common febrile diseases including malaria and dengue fever [4].

The data presented by Farmakiotis et al in their correspondence pertain to nontyphoidal *Salmonella* gastroenteritis, which is pathologically distinct from typhoid fever. Typhoid fever is a severe systemic infection caused by the human-restricted pathogen *S.* Typhi, affecting an estimated 26.9 million people annually, of whom 1% die [5]. In contrast, *Salmonella* gastroenteritis results from a large number of nonhuman restricted *Salmonella* serovars and is self-limiting in nonimmunosuppressed hosts. Eosinopenia is recognized in a variety of infectious diseases and in sepsis syndrome [6, 7]; however the underlying mechanism in infection, and more specifically in typhoid infection, is unclear. In health, eosinophils principally reside at mucosal surfaces including the gut mucosa [8], and it is possible that increased marginalization of these cells during infection at this site may account for the early and marked decrease [9]. These uncertainties highlight the significant gaps remaining in our understanding of both typhoid and other enteric infections as well as more global mechanisms of the mucosal immune response. Both need to be addressed to meaningfully impact the clinical management of patients and vaccination strategies. Human challenge studies provide an ideal setting to enable the detailed investigation of disease and immunobiology, particularly for human-restricted pathogens such as *Salmonella* Typhi [10].

Both our report and those of Farmakiotis and colleagues further highlight the lack of accurate diagnostic tests for typhoid and other *Salmonella* serovars, especially in endemic regions where resources are frequently limited. Prompt diagnosis and treatment of typhoid decreases complications and limits the opportunity for onward transmission [4]. New, reliable diagnostic tests are needed to prevent treatment delays, minimize morbidity and mortality, prevent inappropriate use of antimicrobials, and provide reliable epidemiological data.
